# The Hyperdense Internal Carotid Artery Sign: Prevalence and Prognostic Relevance in Stroke Thrombolysis

**DOI:** 10.4061/2011/843607

**Published:** 2011-08-22

**Authors:** P. R. Fitzsimmons, S. Biswas, A. M. Hill, R. Kumar, C. Cullen, R. P. White, A. K. Sharma, R. Durairaj

**Affiliations:** ^1^Aintree Stroke Centre, University Hospital Aintree, Liverpool L9 7AL, UK; ^2^Department of Radiology, University Hospital Aintree, Liverpool L9 7AL, UK; ^3^Departments of Neurology and Neurosurgery, The Walton Centre NHS Foundation Trust, Liverpool L9 7LJ, UK

## Abstract

*Introduction*. The hyperdense internal carotid artery sign (HICAS) has been suggested as a common marker of terminal internal carotid artery (ICA) thrombus associated with poor outcomes following thrombolysis. We aimed to investigate the prevalence and prognostic significance of the HICAS in an unselected cohort of patients receiving intravenous thrombolysis. *Methods*. Prethrombolysis NCCTs of 120 patients were examined for the presence of the HICAS and hyperdense middle cerebral artery sign (HMCAS). A poor outcome was defined as a discharge Barthel score <15 or inpatient death. *Results*. A HICAS was present in 3 patients (2.5%). Prethrombolysis neurological deficits were significantly more severe in patients with a HICAS (*P* = 0.019). HICAS was not significantly associated with a poor outcome (*P* = 0.323). HMCAS was significantly associated with severe prethrombolysis neurological deficits (*P* = 0.0025) and a poor outcome (*P* = 0.015). *Conclusions*. This study suggests that the prevalence of the HICAS may be lower than previously reported. The presence of a HICAS was associated with severe prethrombolysis neurological deficits in keeping with terminal ICA occlusion. The role of the HICAS as a prognostic marker in stroke thrombolysis remains unclear.

## 1. Introduction

Despite advances in magnetic resonance imaging, noncontrast computed tomography (NCCT) remains the most commonly used imaging modality in acute stroke. Early ischemic changes on NCCT, particularly those in the territory of the middle cerebral artery (MCA), have diagnostic and prognostic implications in patients receiving thrombolytic therapy [1, 2]. However, these changes are often subtle and as such may be of variable reliability [3–6].

The hyperdense middle cerebral artery sign (HMCAS) is a well-established marker of intraluminal MCA thrombus and was amongst the first NCCT changes described in acute ischemic stroke [7]. The presence of a HMCAS is associated with severe initial neurological deficits and poor outcomes following thrombolytic therapy with intravenous recombinant tissue plasminogen activator (rtPA) [8–10]. The persistence of a HMCAS following thrombolysis is also associated with a poor functional outcome [11].

More recently, the hyperdense internal carotid artery sign (HICAS) has been suggested as a common marker of terminal internal carotid artery thrombus [12]. The HICAS has been described as a hyperdensity in the distal segment of the internal carotid artery (ICA) seen on NCCT, which is indicative of thrombus within the supraclinoid segment of the distal ICA. In a previous study of 71 patients receiving intravenous and/or intra-arterial rtPA, a HICAS was present in nearly a quarter of patients and was associated with both a severe initial neurological deficit and a poor outcome [12]. 

We aimed to further investigate the prevalence of the HICAS in a larger, unselected cohort of patients undergoing intravenous thrombolysis. We also aimed to investigate the association of the HICAS with initial severity of neurological deficit and its role as prognostic marker in patients receiving intravenous rtPA.

## 2. Subjects and Methods

Between December 2007 and May 2010, 123 consecutive patients with acute ischemic stroke were treated with intravenous rtPA at Aintree Stroke Centre. Demographic information, stroke risk factors, baseline neurological deficit, and functional outcome at discharge were documented prospectively. For the present study, 120 patients were included. 3 patients were excluded from analysis as they were initially imaged at other centres and as such their prethrombolysis NCCTs were not available for review. All patients received thrombolysis with intravenous rtPA 0.9 milligrams/kg administered in line with standard thrombolysis protocols.

### 2.1. Analysis of Radiological Data

Each patient had a pretreatment NCCT scan and a second scan at 24 hours following thrombolysis. 

30 patients were imaged with a single slice CT scanner, with a section thickness of 4 mm through the posterior fossa and 8 mm for the cerebral hemispheres. 

The remaining 90 patients were imaged by 4- and 16-slice scanners with a section thickness of 2.5 mm through the posterior fossa and 5 mm for the cerebral hemispheres.

All 120 NCCT scans were reviewed retrospectively and independently by a radiologist (SB) and a stroke fellow (PF). Both investigators were blinded to all clinical information and inspected the prethrombolysis NCCT scans for the presence of a HICAS and/or a HMCAS. The HMCAS was defined as “an MCA denser than its contralateral counterpart” [13]. 

**Figure 1:**
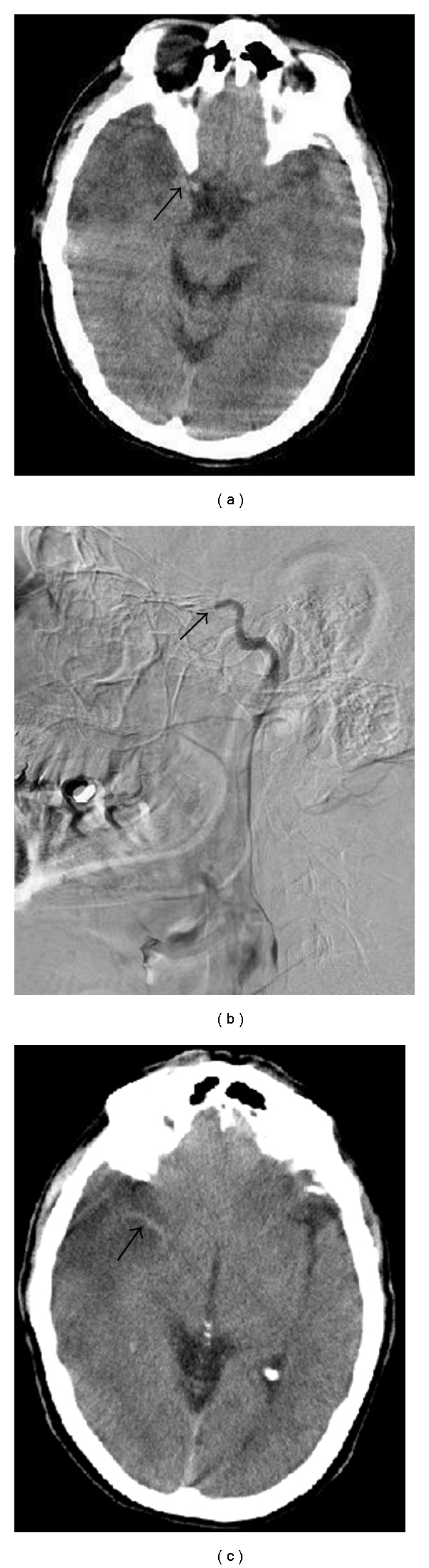
(a) NCCT demonstrating HICAS (arrow). (b) CT Carotid Angiogram demonstrating corresponding terminal carotid occlusion (arrow). (c) NCCT demonstrating HMCAS (arrow).

The HICAS was defined as “A hyperdensity of the supraclinoid part of the ICA observed in the prepontine or premesencephalic cistern where the vessels form the Circle of Willis.” A HICAS was deemed to be present if the distal ICA was denser than its contralateral counterpart [12] (Figure 1). 

Both signs were rated as either present or absent. Disagreements were settled by a third, more experienced investigator (RD), who was also blinded to all clinical data.

### 2.2. Clinical Assessment

Severity of neurological deficits at baseline and 24 hours after thrombolysis were assessed prospectively by using the National Institutes of Health Stroke Scale (NIHSS) conducted by clinicians certified in NIHSS scoring. Patients with NIHSS ≥10 at 24 hours were considered to have severe early postthrombolysis neurological deficits. Barthel scoring was undertaken at discharge as is routine practice in our unit. Poor outcome following thrombolysis was defined as a discharge Barthel score <15 or inpatient death. Haemorrhagic conversion following thrombolysis was defined as any intracranial haemorrhage on NCCT 24 hours after thrombolysis.

### 2.3. Statistical Analysis

Data was analysed in GraphPad Prism 4.0 (GraphPad Software, San Diego, Calif, USA). The D'Agostino-Pearson omnibus test was used to test continuous data for normality. Paired/unpaired *t*-tests were used to compare normally distributed continuous variables with Mann-Whitney *U* tests used to compare nonparametrically distributed continuous data. Fisher's exact test was used to analyse categorical variables. Interobserver agreement for the HICAS and the HMCAS was assessed using Cohen's Kappa. Statistical significance was set at 0.05.

### 2.4. Ethical Review

The project protocol was reviewed by the chair of the local Research Ethics Committee.

## 3. Results

120 patients were assessed. A HICAS was present in 3 patients (2.5%) and absent in 117 (97.5%).  Table 1 demonstrates baseline characteristics and outcomes of patients with a HICAS and without a HICAS. 

In the 3 patients displaying a HICAS the mean maximum density of the affected supraclinoid ICA measured in the Hounsfield units was significantly higher than on the unaffected side. Mean maximum density of the hyperdense ICA 56.3 HU (95% CI 46.3 to 66.4), mean maximum density of the ICA on the unaffected side 28.3 HU (95% CI 26.9 to 29.7), *P* = 0.0067.

A HMCAS was present in 22 patients (18.3%). Patients with a HMCAS showed a nonsignificant trend towards displaying a HICAS (9.1% versus 1%), odds ratio (OR) 9.7 (95% CI 0.834 to 12.3), *P* = 0.086. In the 2 patients who displayed both HICAS and HMCAS, a continuous hyperdensity representing clot was seen between the terminal ICA and MCA. Table 2 demonstrates baseline characteristics and outcomes of patients with a HMCAS and without a HMCAS.

In the patients displaying a HMCAS, the mean maximum density of the affected MCA measured in the Hounsfield units was significantly higher than on the unaffected side. Mean maximum density of the hyperdense MCA 46.3 HU (95% CI 44.0 to 48.6), mean maximum density of the MCA on the unaffected side 35.4 HU (95% CI 32.2 to 38.5), *P* < 0.0001.

### 3.1. Baseline Neurological Deficit and Haemorrhagic Conversion

Patients with a HICAS had a significantly higher baseline mean NIHSS score (Mean NIHSS 21.0 95% CI 16.7 to 25.3) compared to those without this sign (Mean NIHSS 12.8 95% CI 11.7 to 13.9, *P* = 0.019). 

The presence of a HMCAS was also associated with significantly more severe prethrombolysis neurological deficits (Mean NIHSS 16.5 95% CI 14.3 to 18.7) when compared to patients without a HMCAS (Mean NIHSS 12.3 95% CI 11.1 to 13.4, *P* = 0.0025).

Haemorrhagic conversion occurred in 5% of patients, an incidence comparable to that seen in the SITS-MOST monitoring study [14]. No significant associations were observed between the presence of a HICAS (OR 2.45 95% CI 0.11 to 52.7, *P* = 1.0) or a HMCAS (OR 5.0 95% CI 0.94 to 26.7, *P* = 0.074) and intracranial haemorrhage following thrombolysis. 

### 3.2. Short-Term and Long-Term Outcomes Following Thrombolysis

At 24 hours following thrombolysis, severe neurological deficits (NIHSS score ≥10 points) were observed in 2 of 3 patients (66.6%) with a HICAS compared to 44 of 117 patients (37.1%) without a HICAS, OR 3.20 95% CI 0.28 to 36.7, *P* = 0.559.

The presence of a HICAS on prethrombolysis NCCT was not significantly associated with a poor outcome at discharge (Barthel <15 or impatient death). Of the 3 patients with a HICAS, 2 (66%) had a poor outcome, compared with 45 (38.5%) of the 117 patients without HICAS, OR 3.20 95% CI 0.28 to 36.34, *P* = 0.560. 

The presence of a HICAS was not significantly associated with inpatient death (*P* = 1.0).

The presence of a HMCAS on initial prethrombolysis NCCT was significantly associated with both a severe neurological deficit at 24 hours following thrombolysis and a poor outcome at discharge.

A 24-hour NIHSS score of ≥10 was seen in 20 of 22 patients (90.1%) of patients with a HMCAS and 32 of 98 (32.7%) of those without a HMCAS, OR 20.6 95% CI 4.54 to 93.7, *P* < 0.0001.

A poor outcome at discharge was observed in 14 of 22 (63.6%) patients with a HMCAS compared with 33 of 98 (33.7%) without a HMCAS, OR 3.45 95% CI 1.31 to 9.04, *P* = 0.015.

The presence of a HMCAS was not significantly associated with inpatient death (*P* = 0.750).

### 3.3. Interobserver Agreement for the Hyperdense ICA and Hyperdense MCA Signs

Interobserver agreement was excellent for the HMCAS-Cohen's Kappa 0.973 (95% CI 0.919 to 0.999) and fair for the HICAS-Cohen's Kappa 0.275 (95% CI 0.0 to 0.704).

## 4. Discussion

In the first hours following acute ischaemic stroke, NCCT frequently demonstrates early parenchymal changes which may be preceded by hyperdense artery signs suggestive of intraluminal thrombus [15]. These early ischemic changes on NCCT are of prognostic significance in predicting functional outcome before thrombolysis is administered [1, 2]. 

Our study aimed to further investigate the prevalence and prognostic significance of the HICAS. Two previous studies have described distal ICA thrombus resulting in a hyperdense appearance of the terminal ICA on NCCT [12, 16]. Our study contains the largest series of patients to date and is the first study to include an unselected series of patients receiving intravenous thrombolysis with rtPA.

A HICAS was present in 2.5% of the patients included in our study, this did not differ significantly from the 5.8% prevalence reported in the first paper to describe the HICAS [16] but does represent a significantly lower prevalence than the 24% reported in patients receiving thrombolysis by Ozdemir et al. (Fisher's exact test; *P* < 0.0001) [12]. 

The lower prevalence of the HICAS observed in our series of patients undergoing thrombolysis may in part be explained by the NCCT imaging protocols employed. A quarter of the NCCT scans reviewed in our study used a thicker 8 mm slice rather than the 5 mm slices used by Ozdemir et al. [12]. A previous study has demonstrated that a reduction in slice thickness is associated with increased sensitivity in identifying hyperdense intracerebral arteries [16], as such the thicker slices used in some of the scans included in our study may have resulted in false negatives. However, even if these thicker 8 mm slice NCCT are excluded from analysis, the proportion of patients with a HICAS remains significantly lower than that described by Ozdemir et al. [12] and comparable to that observed in the first study to describe the HICAS [16]. 

Our data suggests the prevalence of the HICAS seen on standard prethrombolysis NCCT slices may be lower than that previously reported.

Occlusion of the intracranial ICA is a potentially catastrophic event. When the distal intracranial ICA is acutely occluded patients are frequently refractory to intravenous thrombolysis and the outcome is often poor [17–19]. The significantly higher prethrombolysis NIHSS scores seen in patients with a HICAS in our study reflect the severe neurological deficits seen with terminal ICA thrombotic occlusion and are comparable to the findings of a previous study [12]. 

Unlike the previous study undertaken by Ozdemir et al., we failed to demonstrate a significant association between the presence of a HICAS and severe early post thrombolysis neurological deficits or long term outcomes following thrombolysis [12]. Patients with a HICAS did display nonsignificant trends towards poor outcomes. Due to the low prevalence of the HICAS in our sample, our study lacked sufficient power to accurately determine the prognostic significance of the HICAS and the failure to demonstrate an association between the HICAS and poor outcomes in the present study may be attributable to a type II error. While 2 of the 3 patients with a HICAS failed to make any clinically significant improvement when treated with intravenous rt-PA, 1 of the 3 patients with a HICAS in our study showed an extremely good response to intravenous rt-PA, with an admission NIHSS of 22 decreasing to 8 at 24 hours post thrombolysis. This patients repeat CT scan at 24 hours post thrombolysis demonstrated resolution of the HICAS, suggestive of clot lysis and recanalisation. This patients unusually good response to peripheral rt-PA [17–19] may have contributed a suspected type II error.

Patients with a HMCAS displayed significantly more severe baseline neurological deficits than those patients without this sign. The HMCAS was also associated with poor neurological and functional outcomes at 24 hours and at discharge. The significant associations observed between the HMCAS and both severe baseline neurological deficits and poor outcomes are in keeping with the findings of several previous studies [8–10].

Interobserver agreement was excellent for the HMCAS. Interobserver agreement for the HICAS was only fair; however, this result should be viewed with caution given the low prevalence of the HICAS in our study population, as reflected by the wide confidence intervals seen for Cohen's kappa.

Our study has several limitations. While all clinical data including risk factors, demographics, and outcome following thrombolytic therapy were collected prospectively, prethrombolysis NCCTs were studied retrospectively.

None of the patients included in our study underwent CT angiography prior to intravenous thrombolysis. As such, we are unable to validate the sensitivity and specificity of the HICAS in detecting terminal ICA thrombus against the gold standard of CT angiogram. Validation of presumed thrombotic occlusion against angiographic data may be of particular importance when investigating hyperdense artery signs as vessel calcification resulting in a false-positive HMCAS is well recognised [10, 15, 20]. We do, however note that both studies which have previously described the HICAS found no false positives when the HICAS was validated as a marker of terminal ICA thrombus using CT angiography [12, 16]. This suggests that the HICAS is less prone to false positives attributable to calcified vessels than the HMCAS.

A quarter of the patients included in our study were imaged using 8 mm through the cerebral hemispheres, with the remainder imaged using 5 mm sections. A previous study in nonthrombolysed acute stroke patients has shown that reduction of section thickness from 5 mm to 1 mm improved the sensitivity of detecting intraluminal ICA thrombus from 33% to 100% without reducing specificity. This may account for the low prevalence of the HICAS in our study and the low sensitivity reported in previous studies using 5 mm slices [12, 16]. Future studies using prethrombolysis thin slice NCCT through the course of the ICA as it traverses the prepontine, and premesencephalic cisterns should allow improved sensitivity in detecting distal ICA thrombus and may improve the predictive power of the HICAS as a prognostic marker.

In summary, this study suggests the prevalence of the HICAS on standard prethrombolysis NCCT may be lower than that previously reported. The presence of a HICAS was associated with significantly more severe initial neurological deficits in keeping with acute terminal ICA occlusion. Larger studies, ideally using thin slice CT through the course of the terminal ICA, are required to further investigate the prevalence and prognostic significance of the HICAS in stroke patients receiving thrombolytic therapy.

## Figures and Tables

**Table 1:** Baseline characteristics and outcomes of patients with or without HICAS present.

	Patients with HICAS	Patients without HICAS	
	*n* = 3	*n* = 117	*P*

Age	72.0 (65.4–78.6)	69.7 (67.5–71.9)	0.742
Female	2 (66.6%)	51 (43.6%)	1.0
Baseline NIHSS	21.0 (16.7–25.3)	12.8 (11.7–13.9)	0.019
Time to rtPA (minutes)	176 (37–315)	162 (151–173)	0.626
Atrial fibrillation	1 (33.3%)	27 (23.1%)	0.553
Hyperlipidaemia	2 (66.6%)	42 (35.9%)	0.554
Angina	0 (0%)	20 (17.1%)	1.0
Peripheral vascular disease	0 (0%)	6 (5.1%)	1.0
Hypertension	2 (66.6%)	72 (61.5%)	1.0
Myocardial infarction	0 (0%)	11 (9.4%)	1.0
Diabetes mellitus	0 (0%)	14 (11.9%)	1.0
Current smoker	1 (33.3%)	24 (20.5%)	0.507
Haemorrhagic conversion	0 (0%)	6 (5.1%)	1.0
24 hour NIHSS ≥10	2 (66.6%)	44 (37.1%)	0.559
inpatient death	0 (0%)	19 (16.2%)	1.0
Poor outcome	2 (66.6%)	45 (38.5%)	0.560

Values are mean (95% confidence interval) or *n* (%) as appropriate.

Poor outcome defined as discharge Barthel score <15 or inpatient death.

**Table 2:** Baseline characteristics and outcomes of patients with or without HMCAS present.

	Patients with HMCAS	Patients without HMCAS	
	*n* = 22	*n* = 98	*P*

Age	69.9 (65.1–74.9)	69.7 (67.3–72.1)	0.935
Female	12 (54.5%)	41 (41.8%)	0.344
Baseline NIHSS	16.5 (14.3–18.7)	12.8 (11.1–13.4)	0.0025
Time to rtPA (minutes)	157 (135–180)	165 (153–178)	0.577
HICAS present	2 (9.1%)	1 (1.0%)	0.086
Atrial fibrillation	6 (27.3%)	22 (22.4%)	0.590
Hyperlipidaemia	9 (40.9%)	35 (35.7%)	0.635
Angina	5 (22.7%)	15 (15.3%)	0.526
Peripheral vascular disease	1 (4.5%)	5 (5.1%)	1.0
Hypertension	13 (59.1%)	61 (62.2%)	0.812
Myocardial infarction	1 (4.5%)	10 (10.2%)	0.687
Diabetes mellitus	3 (13.6%)	11 (11.2%)	0.720
Current smoker	6 (27.3%)	19 (19.4%)	0.397
Haemorrhagic Conversion	3 (13.6%)	3 (3.1%)	0.074
24 hour NIHSS ≥10	20 (90.1%)	32 (32.7%)	<0.0001
Inpatient death	4 (18.1%)	15 (15.3%)	0.750
Poor outcome	14 (63.6%)	33 (33.7%)	0.015

Values are mean (95% confidence interval) or *n* (%) as appropriate.

Poor outcome defined as discharge Barthel score <15 or inpatient death.
